# Diversity among principal and GABAergic neurons of the anterior olfactory nucleus

**DOI:** 10.3389/fncel.2014.00111

**Published:** 2014-04-29

**Authors:** Rachel B. Kay, Peter C. Brunjes

**Affiliations:** Department of Psychology, University of VirginiaCharlottesville, VA, USA

**Keywords:** olfactory cortex, olfactory system organization, pyramidal cells, cortical interneurons, GAD67-GFP

## Abstract

Understanding the cellular components of neural circuits is an essential step in discerning regional function. The anterior olfactory nucleus (AON) is reciprocally connected to both the ipsi- and contralateral olfactory bulb (OB) and piriform cortex (PC), and, as a result, can broadly influence the central processing of odor information. While both the AON and PC are simple cortical structures, the regions differ in many ways including their general organization, internal wiring and synaptic connections with other brain areas. The present work used targeted whole-cell patch clamping to investigate the morphological and electrophysiological properties of the AON's two main neuronal populations: excitatory projection neurons and inhibitory interneurons. Retrograde fluorescent tracers placed into either the OB or PC identified projection neurons. Two classes were observed with different physiological signatures and locations (superficial and deep pyramidal neurons), suggesting the AON contains independent efferent channels. Transgenic mice in which GABA-containing cells expressed green fluorescent protein were used to assess inhibitory neurons. These cells were further identified as containing one or more of seven molecular markers including three calcium-binding proteins (calbindin, calretinin, parvalbumin) or four neuropeptides (somatostatin, vasoactive intestinal peptide, neuropeptide Y, cholecystokinin). The proportion of GABAergic cells containing these markers varied across subregions reinforcing notions that the AON has local functional subunits. At least five classes of inhibitory cells were observed: fast-spiking multipolar, regular-spiking multipolar, superficial neurogliaform, deep neurogliaform, and horizontal neurons. While some of these cell types are similar to those reported in the PC and other cortical regions, the AON also has unique populations. These studies provide the first examination of the cellular components of this simple cortical system.

## Introduction

The brain will only be understood once the function of its component circuits and their constituent neuronal elements are unraveled. The astounding diversity of neurons has prompted several attempts at categorization. Perhaps the simplest rubric is to separate neurons into two main groups: excitatory projection neurons, and inhibitory local circuit neurons. However, each of these classes contains broadly different kinds of cells. Indeed, intensive examinations of the neocortex have revealed that excitatory cells can be divided into subtypes based upon their projection targets as well as their physiology and morphology (e.g., Kumar and Ohana, 2008; Brown and Hestrin, 2009; Groh et al., 2010). Inhibitory cells are much more varied and have been categorized on the basis of their morphology, molecular markers, and physiology (McBain and Fisahn, 2001; Markham et al., 2004; Hestrin and Galarreta, 2005; Ascoli et al., 2008). The studies of neocortex have prompted others to investigate whether species of neurons are conserved in other cortical areas including the hippocampus (Price et al., 2005; Klausberger and Somogyi, 2008) and piriform cortex (PC) (Young and Sun, 2009; Suzuki and Bekkers, 2010a,b, 2011; Bekkers and Suzuki, 2013).

The olfactory system is an attractive brain region due to its relative simplicity and high degree of organization. Odor information encoded by sensory neurons in the olfactory epithelium is relayed to the olfactory bulb (OB) where it is processed and then distributed via the lateral olfactory tract to olfactory cortex (Mori and Sakano, 2011). The olfactory cortices are useful models of cortical structure and function as they are not as complex and have fewer layers (2–3) than their six-layered neocortical counterparts. The circuitry of the caudal-most olfactory cortex, PC, has been studied in detail (Neville and Haberly, 2004; Wilson and Sullivan, 2011; Bekkers and Suzuki, 2013). PC includes pyramidal cells as well as several species of interneurons resembling those in neocortex. It is composed of three layers, each made up of a different combination of cells with varying neurochemical phenotypes, electrophysiological properties, and projection targets (Young and Sun, 2009; Suzuki and Bekkers, 2010a,b; Bekkers and Suzuki, 2013).

**Figure 1:**
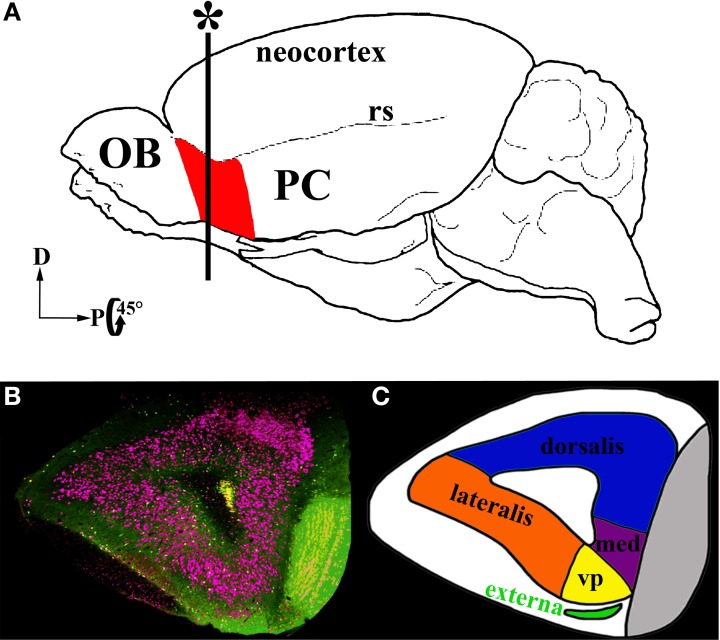
**Location and subdivisions of the AON. (A)** A schematic of a whole mouse brain highlighting the AON (red) in the olfactory peduncle, just posterior to the olfactory bulb (OB) anterior to the piriform cortex (PC) and ventral to the rhinal sulcus (rs). **(B)** Coronal section in the plane marked with an asterisk in **(A)**. GAD67-GFP+ cells: green, Nissl stain: pink. **(C)** A diagram of the subdivisions within the AON. The two major subdivisions are pars externa (green) and pars principalis. Pars principalis is further divided into pars lateralis (orange), pars dorsalis (blue), pars medialis (purple), and pars ventroposterior (yellow).

A second region of the olfactory cortex, the anterior olfactory nucleus (AON)/cortex (Haberly, 2001; Brunjes et al., 2005), has received much less attention. Located between the OB and PC, the AON is the first region to receive input from the OB and is well positioned to influence activity in the entire olfactory circuit (Figure 1A). It provides feedforward projections to PC that synapse on the proximal dendrites of pyramidal cells and thus are aptly positioned to control their activity. The AON also feeds back to every stage of processing in the OB and to the AON in the contralateral hemisphere (Brunjes et al., 2005). The AON and PC share a simple cortical organization with clear lamination and pyramidal cells as their primary output neurons. However, the two regions differ in many ways. For example, the AON is composed of two subdivisions: pars externa, a small band of neurons found at the junction with the OB; and pars principalis, a large ring of cells encircling the olfactory peduncle. Pars principalis is further divided into four subregions based on cardinal directions, ipsilateral and contralateral projections, and cytoarchitecture: pars lateralis, pars dorsalis, par medialis, and pars ventroposterior (Haberly and Price, 1978; Brunjes et al., 2005; Meyer et al., 2006; Illig and Eudy, 2009, Figures 1B,C). Pars principalis has hallmarks of a simple cortex: it has an outer plexiform layer (layer I) and an inner cell zone (layer II) containing pyramidal cells (Brunjes and Kenerson, 2010), a feature that distinguishes it from the trilaminate organization of the PC. The AON and PC also develop in opposing fashions: young neurons populate the superficial layer of the AON before the deep layer, while the reverse is true for the PC. The synaptic connections the two areas have with regions outside the central olfactory circuit also differ (Brunjes et al., 2005). The present study provides the first comprehensive investigation of common cellular subtypes in pars principalis of the AON. The data are important for understanding both the AON's circuitry and for comparing its structure with other forebrain cortical regions. The results indicate that the AON shares cell types with other cortices, reinforcing the notion that it is cortical and not nuclear in nature. The findings also indicate the AON has varieties of neurons not found in the PC, further establishing it as a separate station in olfactory processing.

## Materials and methods

### Animals

All procedures were performing according to NIH guidelines and protocols approved by the University of Virginia IACUC. Animals were housed in standard polypropylene cages with food (8604, Harlan, Frederick MD) and water *ad libitum*. The colony was maintained on a 12:12-h light/dark cycle in a humidity- and temperature-controlled room (22°C). Studies examining the properties of excitatory cells used C57BL/6J mice (Jackson Laboratory, Bar Harbor, ME). Those examining inhibitory cells used hemizygous knock-in GAD67-GFP (Δneo) mice (C57BL6/J background), in which cDNA encoding enhanced GFP was inserted into the GAD67 locus, kindly provided by Dr. Yuchio Yanagawa (Gunma University Graduate School of Medicine; Tamamaki et al., 2003). These hemizygous GAD67-GFP males were mated with C57BL6/J females (Jackson Laboratory, Bar Harbor, ME). GFP-positive pups were identified at birth by visualizing the presence of fluorescent brain tissue through their skulls. Previous studies have indicated that the GAD67-GFP mice have normal behavior and neuroanatomy (Tamamaki et al., 2003). Mice 18–25 days old were used for electrophysiology experiments and 2–3 month olds were used to in immunohistochemistry experiments that surveyed the neurophenotypes of GAD67-GFP cells.

### Projection neuron and inhibitory neuron electrophysiology

In order to visualize projection neurons before targeting for physiology experiments, a retrograde tracer injection was made in C57BL/6J days prior to recording. To visualize and target inhibitory neurons, tissue from GAD67-GFP mice was used for recordings. *Post-hoc* immunohistochemistry was performed on the GAD67-GFP slices to characterize the neurophenotype of the recorded cell.

#### Retrograde tracer injection

Mice (18–23 days old) were anesthetized with isoflurane (2% in oxygen). They were then placed on a heating pad to maintain body temperature and their head secured in a stereotaxic frame (Kopf Instruments, Tujunga, CA). Using aseptic procedures, a midline incision was made, skull landmarks located, and the head leveled to conform to the atlas of Slotnick and Leonard (1975). A small hole was drilled in the skull overlying one of two intended injection sites a) in the middle of the rostro-caudal extent of the OB or b) anterior piriform cortex (APC; 1.75 mm anterior, 3.2 mm lateral to bregma). A glass pipette (tip diameter 30–50 μm) previously filled with fluorescent red microspheres (Lumafluor Inc., Durham, NC) was lowered to the proper depth (~2 and 4 mm respectively). Pressure injections were used to deposit ~300 nl of solution. Mice survived a minimum of 2 days before tissue was taken for slice physiology.

#### Tissue preparation for electrophysiology

Mice (18–25 days old) were deeply anesthetized as above and perfused transcardially with ice-cold carbogenated slicing solution containing the following (in mM): 222 sucrose, 2.5 KCl, 1.5 NaH_2_PO_4_, 2 MgSO_4_-7H_2_O, 27 NaHCO_3_, 2 CaCl_2_. The brain was dissected and the AON cut into 300 μm coronal slices. Subsequently the slices were transferred to a holding chamber at 35°C containing artificial cerebral spinal fluid (ACSF) (in mM): 124 NaCl, 3.5 KCl, 1.5 NaH_2_PO_4_, 2 MgSO_4_-7H2O, 26.2 NaHCO_3_, 10 dextrose, 2.5 CaCl_2_. After 1 h of incubation, the slices were allowed to cool to room temperature. All solutions were continuously bubbled with 5% CO_2_/95% O_2_.

#### Whole-cell patch clamp recordings

Neurons were visualized with fluorescence optics to determine if they were filled with fluorescent microbeads for excitatory cell experiments, or GAD67-GFP for inhibitory neuron experiments, and then targeted using infrared videomicroscopy with a 40X water immersion objective on a Zeiss Axopatch microscope. Patch electrodes were pulled from borosilicate glass and had resistances of 4–6 MΩ when filled with internal solution composed of (in mM): 135 K-methylsulfate, 7 KCl, 0.1 EGTA, 2 Na_2_ATP, 2 MgCl, 0.3 Na_2_GTP, 10 HEPES at pH 7.2, supplemented with 0.2% biocytin. Voltage measurements were not corrected for the calculated liquid junction potential (13.4 mV). A MultiClamp 700B amplifier (Molecular Devices, Union City, CA) was used to obtain whole-cell current clamp recordings from the somata of the identified cells. Capacitance neutralization and bridge balance were carefully adjusted and checked frequently during the experiment. Neurons with high series resistance (>30 MΩ) or unstable recordings were excluded from analysis. During the recording sessions a number of measures were taken. First, a family of evenly spaced current steps (starting from −70 pA, 500 ms duration, 5 s pause between steps, 2–3 repetitions per cell) with amplitudes to 2.5X rheobase was applied to the cell. Rheobase was defined as the amplitude of the smallest depolarizing current that elicited one or more action potentials within the first 300 ms of the current step.

#### Electrophysiology data analysis

Intrinsic membrane properties were calculated using custom routines written in Matlab (MathWorks, Novi, MI). Membrane time constant was obtained by fitting a single exponential to the relaxation of membrane potential (V_m_) following a −70 pA current step. Sag was calculated as (V_m2_ − V_m1_), where V_m2_ was measured at steady state at the end of a −70 pA current step, V_m1_ was measured at the maximal hyperpolarization near the beginning of the step (anti-peak). Single action potential (AP) properties were measured on the first AP that occurred at least 10 ms after the beginning of a current step just above rheobase. Latency to first AP was measured as the time from the beginning of this current step to the peak of the first AP. AP voltage threshold was measured as the V_m_ at which dV_m_/dt first exceeded 40 V/s. AP peak was the voltage reached at the peak of the AP, whereas AP height was the difference between the AP voltage threshold and the peak. AP risetime was the time from AP voltage threshold to the AP peak. AP half-width was defined as the width of the AP halfway between the AP voltage threshold and the peak. The height, risetime, and halfwidth of the afterhyperpolarization (AHP) were calculated for the AHP following the first AP at rheobase, with respect to the AP voltage threshold. A measure of accommodation, the tendency for APs to cluster at the beginning of the step, was quantified as the AP clustering ratio (Chiang and Strowbridge, 2007), defined as the number of APs occurring in the first 100 ms of the step divided by the number of APs during the entire 500-ms step, averaged across all step sizes. The data were analyzed using a tree clustering algorithm with Euclidean distances and Ward's linkage criteria in the statistics toolbox in Matlab.

#### Post-hoc anatomy and immunohistochemistry

At the conclusion of the electrical measurements, the electrode was carefully withdrawn while maintaining the seal. The slice was fixed in 4% paraformaldehyde in phosphate-buffered saline (PBS) for 1 h and then stored in PBS at 4°C until immunostaining. Tissue was incubated in a low concentration of streptavidin-Alexa 350 (Invitrogen, S11249, 1:5000) for 5–10 h in order to identify the cell from which the recording was made. For projection neurons, the tissue was then visualized to confirm the colocalization of the recorded cell with microbeads. For GAD67-GFP cells, slices were run through the same panel of seven antibodies and protocols for two randomly selected primaries at a time as described in the immunhistochemistry section below. After each pair of antibodies, this tissue was imaged on an epifluorescent scope (Zeiss, ImagerA2). Then the same cell was serially stained with another pair of antibodies, until all seven antibodies were tested. Due to the demanding nature of multiple rounds of antibody staining, it is presumed that not all molecular markers present were detected by these methods. After antibody staining, all slices were then processed to visualize dendritic morphology with an Elite ABC kit (Vector Laboratories) at 4°C for 18 h, rinsed, underwent detection by the diaminobenzidine (DAB) reaction, and were mounted and coverslipped. DAB processed cells were reconstructed with Neurolucida (MBF Bioscience). Measurements of soma cross sectional area, the number of dendrites at the soma, the total length of apical and basal dendrites, the number of dendritic branch points, subregional location in the AON, and relative location in regards to the pial surface and anterior limb of the anterior commissure, were collected for all neurons.

### Immunohistochemical survey of GAD67-GFP cells

#### Tissue preparation

Mice (2–3 months old) were used to gather information about the neurophenotypes of GAD67-GFP cells in the AON. The mice were deeply anesthetized with Euthasol (Virbac, St. Louis, MO, 0.39/mg drug/g body weight) and perfused transcardially with 0.1 M phosphate-buffered saline (PBS; pH 7.4) followed by 4% buffered formaldehyde freshly depolymerized from paraformaldehyde. The brains were removed, post-fixed for 4 h and washed in 0.05 M Tris-buffered saline (TBS, pH 7.4). Coronal vibratome sections (~100 μm) of the AON were prepared on a Vibratome, and washed and stored in TBS prior to performing immunohistochemistry.

#### Immunohistochemistry

Double labeling was performed for every possible combination of seven primary antibodies: three calcium binding proteins: calbindin (CB), calretinin (CR), and parvalbumin (PV); and four neuropeptides: cholecystokinin (CCK), neuropeptide Y (NPY), somatostatin (SOM), and vasoactive intestinal peptide (VIP), yielding 21 combinations. Free- floating fixed tissue sections were rinsed in TBS 2 X 10 min, then blocked in TBS plus 1% bovine serum albumin (BSA) and 0.3% Triton X-100 (TX) for 1 h. Sections were then placed in TBS containing two primary antibodies (Table 1) plus 1% BSA and 0.3% TX for 18 h at room temperature on a shaker. Sections were subsequently rinsed (3 × 10 min) and placed with secondary antibodies [donkey anti-mouse Alexa 647; (Invitrogen, Eugene, OR; Catalog number A31571, 1:1000); donkey anti-rabbit Dylight 549 (Jackson ImmunoResearch, West Grove, PA; 711-505-152, 1:1000); donkey anti-rabbit Alexa 647 (Invitrogen, A31573, 1:1000); donkey anti-goat Dylight 549 (Jackson, 705-505-147, 1:1000); donkey anti-goat Alexa 647 (Jackson, 705-605-147, 1:1000)] in TBS plus 1% BSA and 0.3% TX for 2 h. After 3 × 10 min rinses in TBS, the seconds were mounted on slides with SlowFade Gold antifade reagent (Invitrogen, 931317).

**Table 1:** **Primary antibodies used**.

**Antigen**	**Immunogen**	**Manufacturer**	**Catalog/lot number**	**Species**	**Dilution Used**
Calbindin D-28k	Recombinant rat calbindin	Swant (Bellinzona, Switzerland)	300/07 (F)	Mouse monoclonal	1:1000
Calbindin D-28K	Recombinant calbindin	Millipore (Temecula, CA)	AB1778/210710	Rabbit polyclonal	1:2000
Calretinin	Rat calretinin	Millipore (Temecula, CA)	AB1550/JC1597097	Goat polyclonal	1:1000
Cholecystokinin	Gastrin-17	CURE Digestive Diseases Research Center (Los Angeles, CA)	9303	Mouse monoclonal	1:1000
Neuropeptide Y	Neuropeptide Y coupled to bovine thyroglobulin	ImmunoStar (Hudson, WI)	22940/812001	Rabbit polyclonal	1:1000
Parvalbumin	Parvalbumin purified from carp muscle	Swant (Bellinzona, Switzerland)	235/10(F)	Mouse monoclonal	1:5000
Parvalbumin	Rat muscle parvalbumin	Swant (Bellinzona, Switzerland)	PV28/5.5	Rabbit polyclonal	1:1000
Somatostatin	Somatostatin coupled to KLH	ImmunoStar (Hudson, WI)	20067/216002	Rabbit polyclonal	1:5000
Vasoactive intestinal peptide	VIP coupled to bovine thyroglobulin	ImmunoStar (Hundson, WI)	20077/722001	Rabbit polyclonal	1:1000

#### Imaging

For each brain, five sections were chosen from standardized locations: (a) the most anterior section with the AON, (b) the most anterior section without pars medialis, (c) the most anterior section with all subregions of the AON, (d) the most posterior section without tenia tecta, and (e) the most posterior section without PC. Sections were imaged using an Olympus Fluoview confocal microscope using a 20X/0.75NA objective. Images were taken through the entire depth of the specimen at an optical thickness of 3 μm. Individual images were tiled together to reconstruct the entire section. To detect the GFP and the two secondaries, each image was acquired with excitation lasers/ emission filters at 488/505–530 nm, 543/560–615 nm, and 633 nm/low-pass 650 nm. The laser intensity and detector gain were individually adjusted for each filter for maximum dynamic range, then settings were left unchanged for all the images collected in a section.

#### Analysis of antibody labeling

A total of 31,550 GFP+ cells from 105 sections taken from 21 brains were quantified. The number of cells analyzed for one combination of antibodies ranged from 1180 to 1688. For each brain section, GFP+ cells were identified by scanning through all images in each confocal stack. If a GFP+ cell extended through multiple images, it was only measured in the one where it exhibited the highest intensity. To ensure that cells were not overcounted at section boundaries, cells lacking neighboring images on either side, i.e., those located in the top and bottom images of the stack, were not counted. To determine colocalization with other antigens, the mean fluorescence intensity for each GFP+ cell was calculated for each of three wavelengths (488, 543, and 633 nm laser excitation) and confirmed by eye to correspond to antibody signal. Separate background measurements were made for each section for each of the three laser wavelengths by calculating the average intensity of 10 cell-sized areas located in non-cellular regions. Profiles were required to be at least two standard deviations brighter than the background measurement to be included in quantification. This analysis allowed for every GFP+ cell to be categorized as either negative for both antigens, positive for an antigen with a red secondary, positive for an antigen with an infrared secondary, or positive for both antigens. Each cell's location was recorded along three dimensions: location within one of the subregions of the AON, location along an anterior-posterior dimension, and the location along a deep to superficial dimension. The point between superficial and deep regions was determined by measuring half the distance from the outside of layer I and the fiber bundle in the center of the AON, the anterior limb of the anterior commissure.

For the production and analysis of figures, images were acquired and minimally adjusted for brightness and contrast with Adobe Photoshop CS5 and plates were constructed with Adobe Illustrator (San Jose, CA).

## Results

Despite its large size and central location in the olfactory circuit, relatively little is known about the neuronal elements that make up the AON. While a few studies have examined cells based on morphology, electrophysiology, or neurochemistry (Lei et al., 2006; Brunjes and Kenerson, 2010; Brunjes et al., 2011; McGinley and Westbrook, 2011; Markopoulos et al., 2012), none have examined all sets of parameters within the same neurons. Roughly 88% of the volume and 78% of the neurons in the AON are in pars principalis (Brunjes et al., 2011); as a result this region was chosen for examination.

The present study used whole-cell patch clamping to record the electrical properties of individual cells, followed by immunohistochemistry to identify molecular phenotypes and then morphological reconstruction. The data indicates that the AON contains two varieties of pyramidal cells, and five distinct interneuronal types. Organized by morphology, the following sections describe the electrophysiological and molecular phenotypes and locations of these cell classes in the AON.

### Pyramidal cells

**Figure 2:**
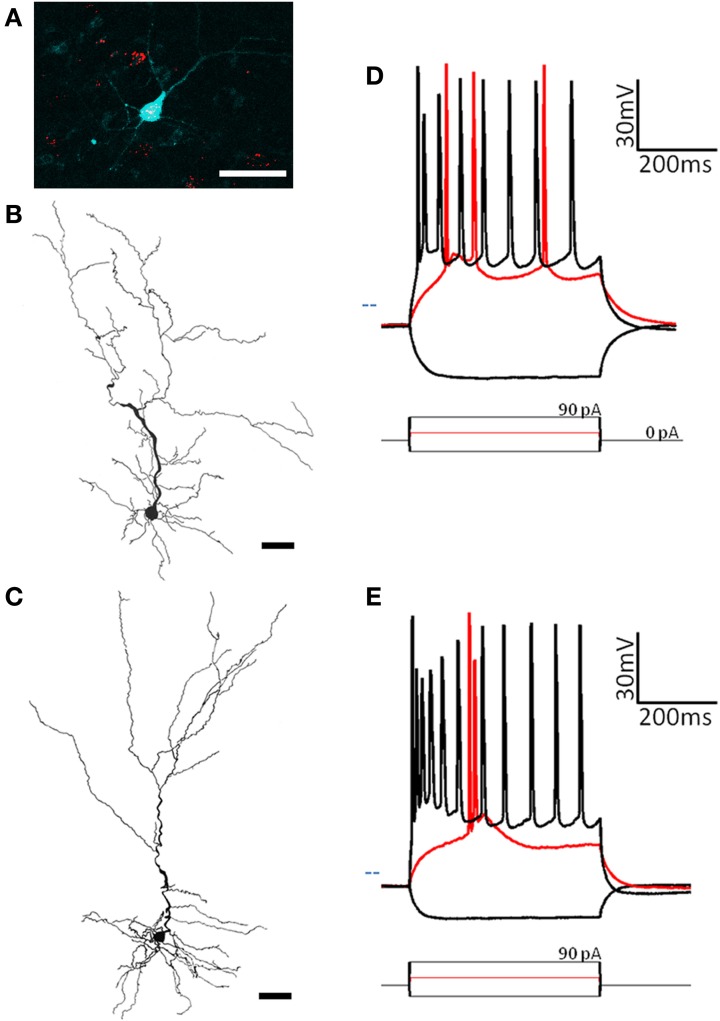
**Two subtypes of pyramidal cells are found in pars principalis. (A)** Cells containing red microbeads from a tracer injection into the olfactory bulb, and the pyramidal cell targeted for recording and filled with biocytin (blue). Scale bar = 50 μm. **(B,C)** Cameralucida drawings of a typical superficial pyramidal cell **(B)** and a typical deep pyramidal cell **(C)** show similar morphologies. Scale bar = 100 μm. **(D,E)** Voltage responses (top) for three current steps (bottom) in a typical superficial pyramidal **(D)** and a deep pyramidal cell **(E)**. Red traces are at rheobase. Dotted line indicates transmembrane voltage of −60 mV.

Pyramidal cells have a uniform morphology that has been well characterized (Brunjes and Kenerson, 2010). Typical of most regions, pars principalis pyramidal neurons have a large triangular shaped cell body with one or two large spiny apical dendrites directed toward the pial surface, and several smaller spiny basilar dendrites (Figures 2B,C). The electrophysiological properties of AON pyramidal neurons in pars lateralis have been reported to be similar to but slightly different from neighboring brain regions (McGinley and Westbrook, 2011). Many pyramidal cells send excitatory projections to areas in the olfactory circuit including the OB and PC (Haberly, 2001; Brunjes et al., 2005; Markopoulos et al., 2012). The present study characterized pyramidal neurons based on their projection target, subregional location, deep to superficial location within layer II, and electrophysiological properties.

#### Projection target

By injecting fluorescent retrograde tracers into either the OB or PC, we could select cells with known projections and visualize them for recording (Figure 2A). The sample contained 19 cells projecting to the ipsilateral OB, six to the contralateral OB, six to the ipsilateral PC, and three to the contralateral PC. When grouped by their projection target, no significant differences were found for any of the 17 electrophysiological properties or four morphological properties measured [Table 2, ANOVA, *F*_(3, 29)_ ≤ 0.55, *p* = ns].

**Table 2:**
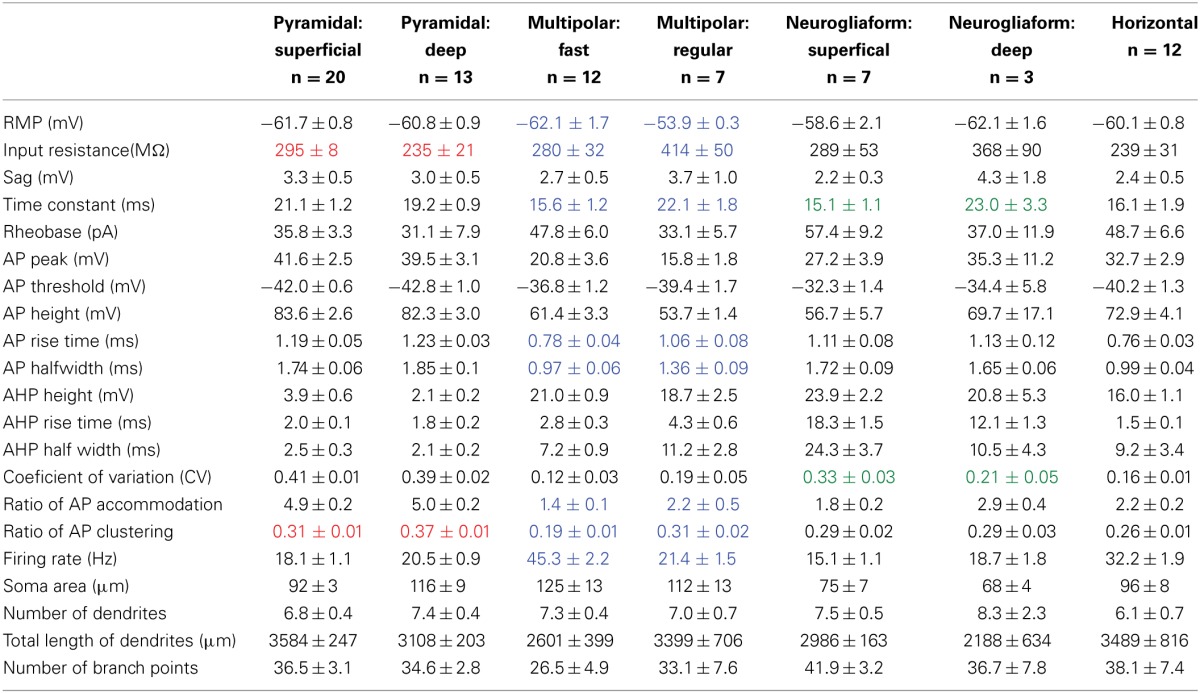
**Electrophysiological and morphological properties of seven cell classes**.

^*^Colored numbers indicate a significant different between groups, p < 0.05.

#### Subregion of pars principalis

The projection neurons from the sample had somas located in one of three subregions of pars principalis: pars lateralis (*n* = 12), pars dorsalis (*n* = 12), and pars medialis (*n* = 9). Pyramidal neurons in pars medialis have a significantly smaller total length of dendrites (2621 μm ± 92) as compared to cells in pars lateralis [3814 μm ± 301; ANOVA, *F*_(2, 30)_ = 4.97, *p* < 0.05]. All other parameters measured did not differ [ANOVA, *F*_(2, 30)_ = 2.05, *p* = ns].

#### Deep/superficial location projection target

As well as their location within subregions of the AON, the location of each cell was categorized as either superficial (top half of layer 2 and therefore closer to the pial surface) or deep (bottom half of layer 2 and therefore closer to the anterior limb of the anterior commissure, the inner white matter region of the AON). Substantially different electrical properties were observed between superficial (*n* = 13; Figure 2D, Table 2) and a deep (*n* = 20, Figure 2E, Table 2) projection neurons. Deeper cells had significantly lower input resistance compared to superficial cells [235 MΩ ± 21 vs. 295 MΩ ± 8; *t*_(31)_ = 3.04, *p* < 0.01] as well as higher action potential clustering ratios [0.37 ± 0.01; 0.31 ± 0.01; *t*_(31)_ = 3.20, *p* < 0.01] reflected by the larger number of action potentials at the beginning of the current step in (Figures 2D,E).

#### Comparison with GAD67-GFP cells

Pyramidal cells, regardless of their location, had a number of characteristics that set them apart from the other cells examined: (1) All pyramidal cells fired either a double or triple action potentials at rheobase (Figures 2D,E). (2) The clustering of action potentials at the beginning of the current step was more pronounced in the deep cells, but the clustering ratio for even the superficial cells was significantly higher than other non-clustering cell types in our study [pyramidal cells: 0.31 ± 0.01; MP fast: 0.19 ± 0.01; ANOVA, *F*_(6, 67)_ = 16.89, *p* < 0.001]. (3) The action potential heights for both classes of pyramidal neurons (deep: 39.5 mV ± 3.1; superficial: 41.6 mV ± 3.1) were significantly higher than all but one of the other cell types examined [horizontal: 72.9 mV ± 4.1; ANOVA, *F*_(6, 67)_ = 8.27, *p* < 0.001]. (4) The pyramidal cells also had a significantly smaller afterhyperpolarization height (deep: 2.1 mV ± 0.2; superficial: 3.9 mV ± 0.6) and afterhyperpolarization halfwidth (deep: 2.1 ms ± 0.2; superficial: 2.5 ms ± 0.3) when compared to any of the other cell types studied [ANOVA, height: *F*_(6, 67)_ = 9.39, *p* < 0.001; halfwidth: *F*_(6, 67)_ = 12.52, *p* < 0.001]. Pyramidal cells in the AON therefore comprise a distinct neuronal class with deep and superficial subclasses.

### Non-pyramidal, GAD67-GFP cells

The present study employed a transgenic mouse in which GABA synthesizing (GAD67) cells produce a green fluorescent protein (GFP) in order to target inhibitory neurons in the AON. We first characterized these GAD67-GFP neurons by examining the proportion that express molecular markers subdividing subspecies of GABAergic cells. Several different subtypes of inhibitory cells in the AON were observed that are differentially distributed in the tissue. Next, we targeted individual GAD67-GFP cells to measure their electrophysiological, morphological, neurochemical properties. The results indicate that pars principalis contains at least five distinct classes of inhibitory neurons.

#### Distribution of molecular markers

**Figure 3:**
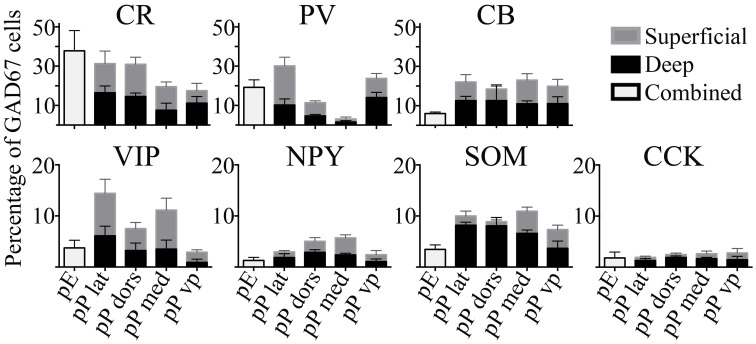
**The percentages of GAD67 cells that colocalize with the seven molecular markers tested vary by region.** Labeled cells in the AON subdivisions [pars externa (pE), pars lateralis (pP lat), pars dorsalis (pP dors), pars medialis (pP med), pars ventroposterior (pP vp)] were quantified as a percentage of all the GAD67+ cells in the region. For subregions of pars principalis, superficial area of the sample is shown as light gray bars, and the deep area as black.

The seven molecular markers examined (CB, CR, PV, VIP, SOM, NPY, CCK) had varying levels of expression in pars principalis, as well as differences based on subregion and deep to superficial location (Figure 3). Averaged across subregions, the three calcium-binding proteins (CR, PV, and CB) were expressed in larger proportions of GAD67-GFP cells (17–25%) than the neuropeptides (3–9%). Five of the molecular markers exhibited different expression based on location. Within pars principalis there was (a) a smaller proportion of GAD67-GFP cells containing PV in pars medialis than in the other subregions [3.0% ± 1.9; ANOVA, *F*_(3, 8)_ = 12.2, *p* < 0.01] and (b) a greater number of SOM immunoreactive cells in the deeper proportion of pars lateralis and pars dorsalis when compared to the superficial proportion [deep lateralis: 5.1% ± 0.5; superficial lateralis: 1.3% ± 0.9; *t*_(4)_ = 3.8, *p* < 0.05; deep dorsalis: 5.0% ± 1.5; superficial lateralis: 0.3% ± 0.9; *t*_(4)_ = 3.0, *p* < 0.05]. No significant differences in the number of cells labeled by the seven probes were found based on anterior-to- posterior location. The expressions of these markers show pars principalis to be a heterogeneous structure that differs by both by subregion and deep-to-superficial location.

**Figure 4:**
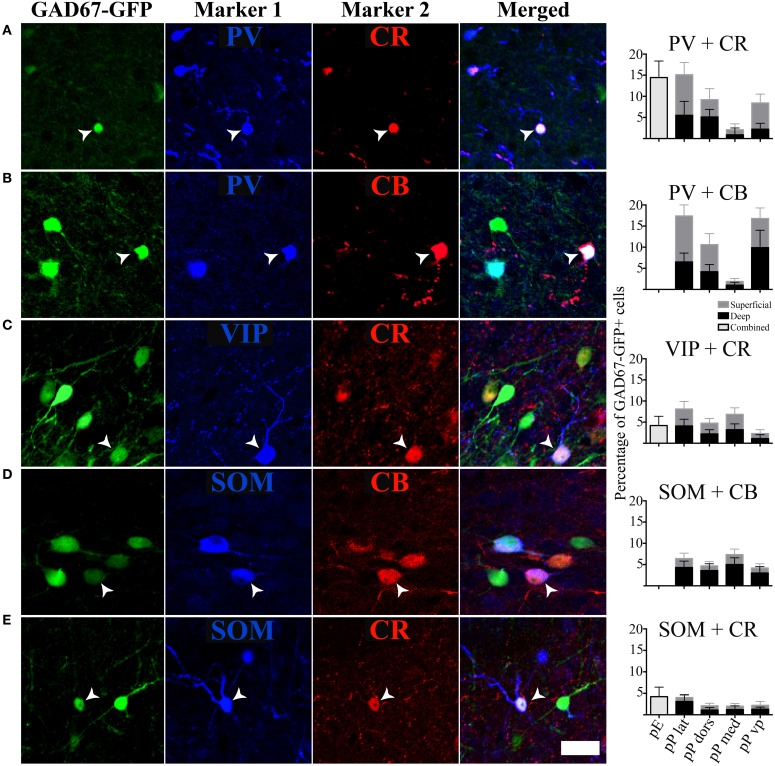
**Specific pairs of antigens colocalize with GAD67. (A–E)** Photomicrographs of the GAD67-GFP cells stained for an additional two markers. White arrows point to cells with triple colocalization. Far right column: quantification of the percentage of GAD67 cells colocalized with the pairs of molecular markers in the different subregions of the AON, [pars externa (pE), pars lateralis (pP lat), pars dorsalis (pP dors), pars medialis (pP med), pars ventroposterior (pP vp)]. Superficial area of the sample is shown as light gray bars, and the deep area as black.

**Figure 5:**
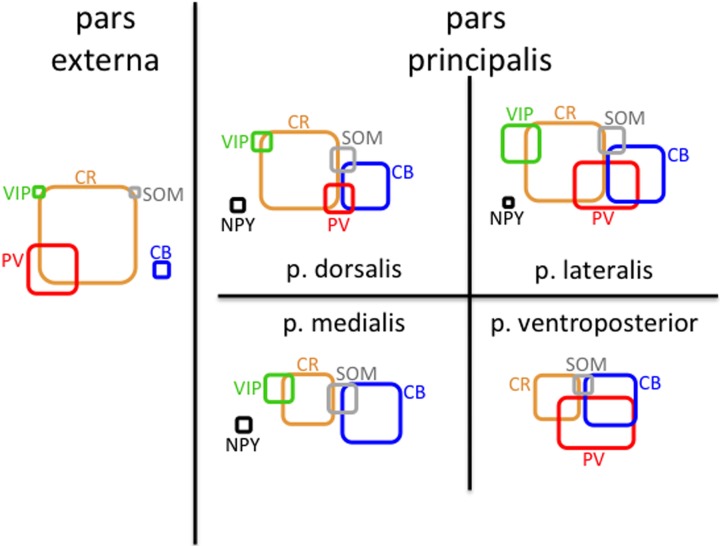
**Schematic of molecular marker expression is variable by subregion of the AON.** Rounded rectangles are scaled so their areas represent the density of cells (mm^3^) that are positive for the indicated molecular marker in that region. The amount of overlap represents the approximate fraction of cells that were double-labeled for the indicated markers.

In addition to analyzing the distribution of GAD67-GFP neurons expressing a single type of molecular marker, we further sought to classify neurons based on their coexpression of two different markers. We tested all 21 pairwise combinations of the seven molecular markers. The results are shown by AON subregion in Figures 4, 5 summarizes the main features of the data. Of the 21 possible pairs of markers, six combinations were found within the AON: CR + PV, CR + VIP, CR + SOM, CB + PV, CB + SOM, VIP + CCK). Of these combinations two had subregional differences in pars principalis: (1) CR+ PV cells were more numerous in pars lateralis [15.1; ± 3.4 ANOVA, *F*_(3, 8)_ = 3.2, *p* < 0.05], and (2) VIP + CCK cells were not found in pars medialis or pars ventroposterior. Once again, the differential expression of combinations of markers confirms that the AON contains a complex variety of inhibitory neurons that vary in their expression by subregion. The experiments that follow investigate the electrical and morphological properties of these GAD67-GFP cells, with additional measures taken to compare molecular markers with physiological and morphological profiles.

#### Multipolar cells

**Figure 6:**
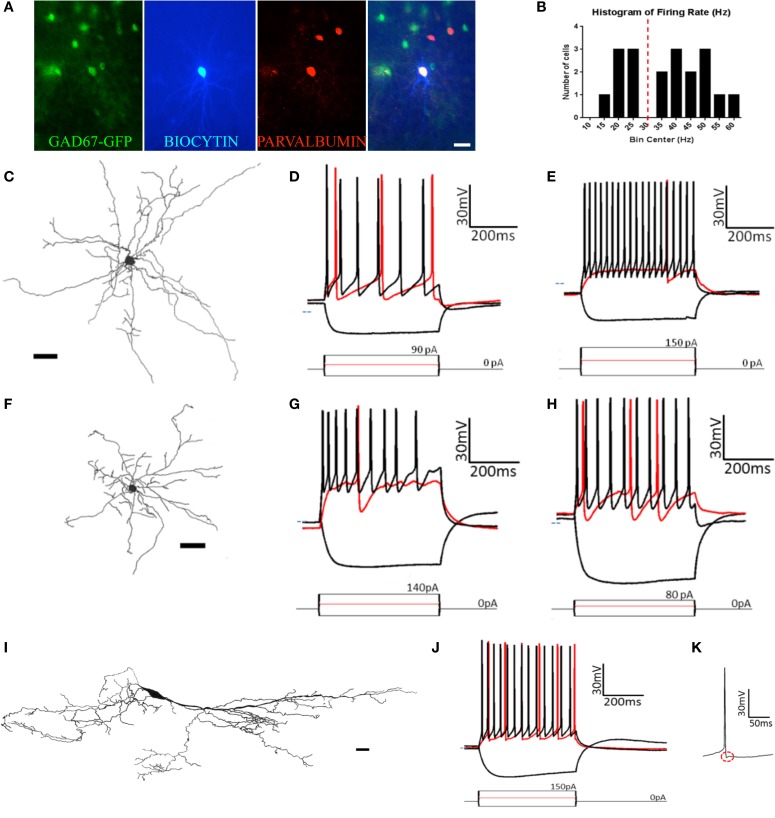
**At least five types of non-pyramidal cells are found in pars principalis. (A)** *Post-hoc* immunohistochemistry of a GAD67-GFP+ multipolar cell filled with biocytin after patch-clamp recording and subsequently stainined for parvalbumin. The merged image (far right) confirms the multipolar cell to be GAD67+ and PV+. Scale bar = 100 μm. **(B)** Histogram of the bimodally distributed firing rates of multipolar cells in the sample. Dashed red line shows 30 Hz as the boundary between regular-spiking and fast-spiking. **(C,F,I)** Cameralucida drawings of a typical **(C)** multipolar cell, **(F)** neurogliaform cell, and **(I)** horizontal cell. Scale bars = 100 μm. **(D,E,G,H,J)** Voltage responses (top) for three current steps (bottom) in a typical **(D)** regular-spiking multipolar cell, **(E)** fast-spiking multipolar cell, **(G)** superficial neurogliaform cell, **(H)** deep neurogliaform cell, and **(J)** horizontal cell. Red traces are at rheobase. Dotted line indicates transmembrane voltage of −60 mV. **(K)** A single action potential of the horizontal cell in **(J)** at a slower timescale. The red circle highlights the “notch” in the afterhyperpolarization.

Unlike pyramidal cells, which have dendrites only at apical and basilar regions, multipolar cells have widely branching aspiny dendrites extending from all regions of the soma (Figure 6C). The multipolar cells examined were located in one of three regions of pars principalis: pars lateralis (*n* = 12), pars dorsalis (*n* = 3), and pars medialis (*n* = 4). None of the 17 electrophysiological and 4 morphological parameters measured differed based on location [ANOVA, *F*_(2, 16)_ ≤ 3.067, *p* = ns]. The firing rates of the multipolar cells were bimodally distributed (Figure 6B). As a result, the sample was subsequently divided into fast (>30 Hz, *n* = 12) and regular (<30 Hz, *n* = 7) spiking cells (Table 2, Figures 6D,E). The two groups differed on a number of measures. Regular firing cells had lower resting membrane potentials [regular: −53.9 mV ± 0.3; fast: −62.1 mV ± 1.7; *t*_(17)_ = 3.50, *p* < 0.01], higher input resistances [regular: 414 MΩ ± 50; fast: 280 MΩ ± 32; *t*_(17)_ = 2.40, *p* < 0.05], longer time constants [regular: 22.1 ms ± 1.8; fast: 15.6 ms ± 1.2; *t*_(17)_ = 3.20, *p* < 0.01], longer action potential risetimes [regular: 1.06 ms ± 0.08; fast: 0.78 ms ± 0.04; *t*_(17)_ = 3.40, *p* < 0.01], longer action potential halfwidths [regular: 1.36 ms ± 0.09; fast: 0.97 ms ± 0.06; *t*_(17)_ = 3.91, *p* < 0.01], more action potential accommodation [regular: 2.2 ± 0.5; fast: 1.4 ± 0.1; *t*_(17)_ = 2.13, *p* < 0.05], and more action potential clustering [0.31 ± 0.02; 0.19 ± 0.01; *t*_(17)_ = 5.23, *p* < 0.001].

Eight of the 12 fast spiking and three of the seven regular firing multipolar cells were located in the deep portion of the AON. Due to the larger proportion of deep cells being fast spiking, there were differences between deep and superficial cells. However, when separated by firing rate there was no difference between the deep and superficial cells for either fast spiking [*t*_(10)_ ≤ 0.97, *p* = *ns*] or regular spiking [*t*_(5)_ ≤ 0.67, *p* = *ns*].

Although the molecular phenotype could not be determined for all the multipolar cells, 11 of the 18 were found to be immunopositive for one of the seven molecular markers examined. Of the 12 fast spiking cells, five were immunopositive for CB and four for PV (Figure 6A). Of the seven regular firing cells two were immunopositive for CB.

#### Neurogliaform cells

Similar to multipolar cells, neurogliaform cells have dendrites arising from many points on the soma. Unlike multipolar cells, their cell bodies are smaller and their dendrites are relatively short (Figure 6F). The dendrites have a large number of branches and form a web around the cell. Neurogliaform cells have smaller cell somas than any other cell types [cell area: 72.8 μm^2^ ± 5.1; ANOVA, *F*_(3, 50)_ = 5.20, *p* < 0.01].

The neurogliaform cells in our sample were located in pars lateralis (*n* = 2), pars dorsalis (*n* = 4), and pars medialis (*n* = 4). There were no significant differences for any of the parameters measured in regards to subregion of the AON [ANOVA, *F*_(2, 7)_ ≤ 1.28, *p* = ns]. Neurogliaform cells were found in both the deep (*n* = 3) and superficial (*n* = 7) portions of the AON. Superficial neurogliaform cells had shorter time constants (15.1 ms ± 1.1) than deep cells [23.0 ± 3.3; *t*_(8)_ = 2.94, *p* < 0.05]. Superficial cells also have more variation in their firing rate, measured by a coefficient of variation [superficial: 0.33 ± 0.03; deep: 0.21 ± 0.05; *t*_(8)_ = 2.39, *p* < 0.05; Table 2]. Unlike multipolar cells, all of the 10 neurogliaform cells in our sample all were immunonegative for the seven molecular markers tested.

The firing pattern of neurogliaform cells was characterized by a slow rise to depolarization followed by a long action potential and a long and large afterhyperpolarization (Figures 6G,H). The action potential threshold for both superficial and deep neurogliaform cells was less negative than other cell types [superficial: −32.3 mV ± 1.4; deep: −34.4 mV ± 5.8; ANOVA, *F*_(6, 67)_ = 8.08, *p* < 0.001]. The action potential halfwidth was longer than the other non-pyramidal cell types [superficial: 1.72 ms ± 0.09; deep: 1.65 ms ± 0.06; ANOVA, *F*_(6, 67)_ = 25.29, *p* < 0.001]. Both the superficial and deep neurogliaform cells had afterhyperpolarization rise times at least three times longer than the other cell types [superficial: 12.1 ms ± 1.3; deep: 18.3 ms ± 1.5; ANOVA, *F*_(6, 67)_ = 144.7, *p* < 0.001].

#### Horizontal cells

Horizontal cells have a large ovoid cell body with spiny dendrites extending from nearly opposite poles and along the polar plane (Figure 6I). Classic horizontal cells in the AON have been described in layer 1 (Brunjes and Kenerson, 2010).

The horizontal cells in our sample were found in either pars lateralis (*n* = 9) or pars dorsalis (*n* = 3). There were no differences in electrical or morphological properties based on subregion [*t*_(10)_ ≤ 1.215, *p* = *ns*]. The cell bodies of the horizontal cells in our sample were located in either in layer 1 (*n* = 8) of pars principalis or in the superficial portion of layer 2 (*n* = 4). There were no differences found based on location [*t*_(10)_ ≤ 1.66, *p* = *ns*]. Interestingly, three of the cells in our sample were not oriented horizontally to the pial surface. Rather these cells had their somas located in layer two and their dendrites oriented vertically projecting both to the deep portions of layer 2 and the superficial portions of layer 1. The morphological and electrophysiological properties of these “vertical” horizontal cells were indistinguishable from regular horizontal cells [*t*_(10)_ ≤ 1.33, *p* = *ns*]. Of the 12 horizontal cells in our sample, all 12 were immunonegative for the seven markers tested.

Horizontal cells have a distinct firing pattern (Figure 6J; Table 2). After the peak of the afterhyperpolarization, these cells had a biphasic return to resting membrane potential characterized by first fast and then slower rates of change. The two-stage rise creates a unique “notch” at every afterhyperpolarization (Figure 6K). Additionally, horizontal cells have a shorter action potential rise time (0.76 ms ± 0.03) and action potential halfwidth (0.99 ms ± 0.04) when compared to all other cell types except the fast spiking multipolar cells [risetime: ANOVA, *F*_(6, 67)_ = 13.66, *p* < 0.001; halfwidth: ANOVA, *F*_(6, 67)_ = 25.29, *p* < 0.001]. The firing rate for horizontal cells (32.2 Hz ± 1.9) is not as fast as fast spiking multipolar cells (45.3 Hz ± 2.2), but it is faster than all of the other cell types [ANOVA, *F*_(6, 67)_ = 45.00, *p* < 0.001].

#### Cluster analysis of seven cell types in pars principalis

**Figure 7:**
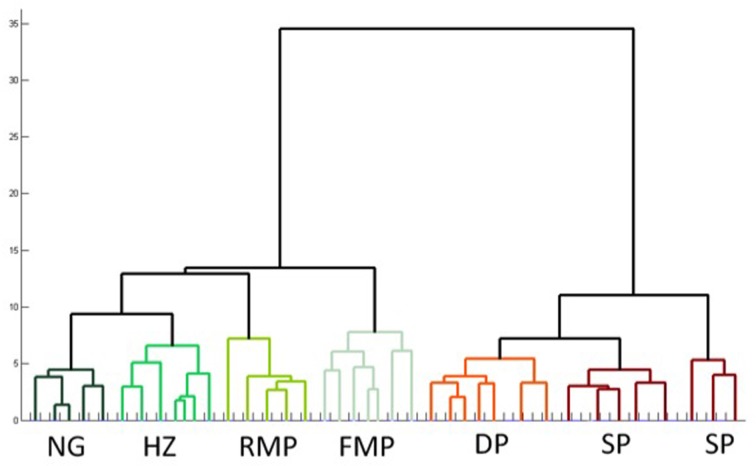
**Unsupervised hierarchical cluster analysis confirms subtypes of cells in pars principalis.** The analysis used all 17 electrophysiological and four morphological properties to link similar cells. NG, neurogliaform; HZ, horizontal; RMP, regular multipolar; FMP, fast multipolar; DP, deep pyramidal; SP, superficial pyramidal.

Cluster analysis, an automated procedure that identifies related groups of objects in a sample, is often used to confirm cell classification schemes (Tamas et al., 1997). In the data presented above, the sample of 74 cells was grouped into 7 classes based on morphology and physiology: superficial pyramidal, deep pyramidal, fast spiking multipolar, regular spiking multipolar, superficial neurogliaform, deep neurogliaform, and horizontal. These distinctions were confirmed by an unsupervised hierarchical cluster analysis using the 21 physiological and morphological cell properties. The dendrogram (Figure 7) shows an overall organization into the 7 classes with only four neurons that did not cluster into their preassigned groups. Interestingly, superficial pyramidal cells clustered into two distinct groups, while neurogliaform cells did not cluster into deep and superficial groups.

### Observations on pars externa

The structure, function and connectivity pars externa differs substantially from pars principalis (Brunjes et al., 2005; Kikuta et al., 2010; Kay et al., 2011). It has received relatively little study and little is known about interneuron populations in the region. Targeting individual cells is challenging as the region is much smaller, thinner, and contains tightly packed cells (Brunjes et al., 2011). While pars externa was not a primary focus of this investigation, two observations were made that suggest the region would be interesting for further study.

**Figure 8:**
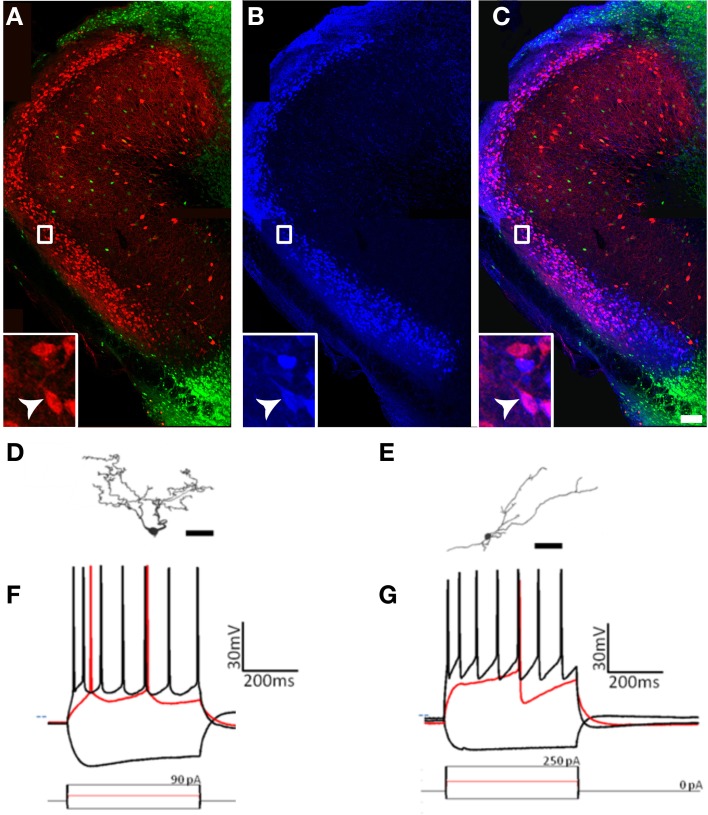
**Cells of pars externa have unique molecular and physiological properties. (A–C)** Calbindin-positive, GAD67-negative cells in pars externa project to the contralateral olfactory bulb. **(A)** GAD67-GFP cells (green) do not colocalize with the large, dense population of calbindin (red) positive cells in pars externa. **(B)** Pars externa cells that project to the contralateral olfactory bulb (blue) are visualized 3 days after a retrograde tracer injection. **(C)** Merged image of **(A)** and **(B)** shows many, but not all of the pars externa cells projecting to the contralateral bulb to colocalize with calbindin (white arrow, inset). Scale bar = 200 μm. **(D–G)** A small sampling of pars externa cells show various phenotypes. **(D,E)** Cameralucida drawings of two pars externa neurons. Scale bars = 100 μm. **(F,G)** Voltage responses (top) for three current steps (bottom) in the cells shown in **(D,E)**, respectively. Red traces are at rheobase. Dotted line indicates transmembrane voltage of −60 mV.

First, the expression of molecular markers that colocalized with GAD67-GFP differed substantially from pars principalis (Figure 8). Pars externa had a smaller proportion of GAD67-GFP cells expressing CB [5.9% ± 0.8; ANOVA, *F*_(4, 10)_ = 3.6, *p* < 0.05], SOM [0.6% ± 0.6; ANOVA, *F*_(4, 10)_ = 6.2, *p* < 0.01], VIP [3.7% ± 1.5; ANOVA, *F*_(4, 10)_ = 4.6, *p* < 0.05], or NPY when compared to pars dorsalis or pars medialis [externa: 1.3% ± 0.6; dorsalis: 5.1% ± 0.2; medialis: 5.7% ± 0.4; ANOVA, *F*_(4, 10)_ = 8.6, *p* < 0.01]. Unlike pars principalis, PV + CB, CB + SOM, or VIP + CCK, containing cells were not found in pars externa. CR + PV cells were more numerous in pars externa than in pars dorsalis or medialis [externa: 18.4% ± 4.5; pars principalis: dorsalis: 10.6% ± 0.7; medialis: 0.8% ± 0.5; ANOVA, *F*_(4, 10)_ = 8.4, *p* < 0.01]. Pars externa also contained a region dense with CB-positive cells that did not colocalize with GAD67 (Figure 8A). Retrograde tracer (BDA) injections into the OB revealed that many externa cells projecting to the contralateral OB are CB-positive and GAD67-negative (Figures 8A–C). No GAD67-positive cells projecting to the OB were observed.

Second, while some of the cells observed had physiological signatures similar to those seen in pars principalis [for example, spiny cells (Figure 8D) with firing patterns similar to pars principalis pyramidal cells (Figure 8F), and non-spiny cells (Figure 8E) with firing patterns similar to regular multipolar cells (Figure 8G)], the sample also contained novel neurons. Among these were a single-spiking unipolar cell, a fast adapting unipolar GAD67-GFP cell, a non-adapting pyramidal-like cell, and an irregularly firing bitufted cell. Although more research is needed, these preliminary findings suggest much diversity in the cells of pars externa.

## Discussion

The work outlined above presents the first classification of cells in the AON based upon morphology, electrophysiology, and molecular phenotype. Two types of projection neurons: deep and superficial pyramidal cells (Figure 2) were identified. Five subtypes of GABAergic cells were recognized based upon firing patterns and morphology: multipolar fast spiking, multipolar regular firing, neurogliaform superficial, neurogliaform deep, and horizontal cells (Table 3). Each of these classes is examined in more detail below. Additionally, preliminary evidence is provided indicating that cell types in pars externa share both similarities and remarkable differences with those in pars principalis.

**Table 3:** **Cell types in other brain regions most similar to pars principalis**.

**Pars principalis**	**Piriform cortex**	**Hippocampus**	**Neocortex**
**Superficial pyramidal**	**Semi-lunar and superficial pyramidal^a^**	**Non-bursting pyramidal^b^**	**Regular-firing, non-bursting pyramidal^c^^,^^d^**
Layer II	Layer II	Stratum pyramidale	Layers II- VI
*Categorized by deep/superficial location and firing pattern*	*Categorized by deep/superficial location, morphology and firing pattern*	*Categorized by laminar location and firing pattern, CB+*	*Categorized by laminar location, morphology, projection target, and firing pattern*
**Deep pyramidal**	**Superficial pyramidal^a^**	**Non-bursting pyramidal^b^**	**Regular-firing, non-bursting pyramidal^c^^,^^d^**
Layer II	Layers II, III	Stratum pyramidale	Layer II-VI
*Categorized by deep/superficial location and firing pattern*	*Categorized by deep/superficial location, morphology and firing pattern*	*Categorized by laminar location and firing pattern, CB+*	*Categorized by laminar location, morphology, projection target, and firing pattern*
**Fast-spiking multipolar**	**Fast-firing multipolar^e^**	**Fast-spiking basket^f^**	**Large fast-spiking basket^g^**
Layer II	Layers IIb and III	Stratum pyramidale	Layers II-VI
*Found deep and superficial CB+ or PV+*	*Only in deeper regions CB+ and/or PV+*	*PV+*	*PV+*
**Regular-spiking multipolar**	**Regular-firing multipolar^e^^,^^h^**	**CA1 oriens lacunosum-moleculare interneuron^f^**	**Classic non-accommodating Martinotti cells^i^**
Layer II	Layers IIb and III	Stratum oriens	Layers II-VI
*Found deep and superficial CB+*	*Only in deeper regions SOM+ and/or CB+*	*SOM+*	*SOM+*
**Superficial neurogliaform**	**Neurogliaform^g^**	**Neurogliaform^f^^,^^j^**	**Neurogliaform^i^**
Layer II	Layer IIa	Stratum lacunosum–molecular	Layers II/III, IV
*More superficial corresponds to more irregular firing*	*More superficial corresponds to more irregular firing*	*intermittent and regular-firing subtypes*	*Intermittent, and regular-firing subtypes*
*Immunonegative*	*Immunonegative*	*NPY+*	*Very small proportion of non-pyramidal cells*
			*Immunonegative*
**Deep neurogliaform**	**Neurogliaform^g^**	**Regular-firing neurogliaform^f^^,^^j^**	**Regular-firing neurogliaform ^i^**
Layer II	Layers IIb, III	Stratum lacunosum-molecular	Layers II/III, IV
*Immunonegative*	*Immunonegative*	*NPY*+	*Very small proportion of non-pyramidal cells*
			*Immunonegative*
**Horizontal**	**Horizontal^g^**		**Cajal Retzius**
Layer I	Layer I		Layer I
*Vertically oriented subtypes, immunonegative*	*All horizontally oriented, immunonegative*		*Only present in immature tissue (<P10 in rats)*

a Suzuki and Bekkers (2011),

b Hemond et al. (2008),

c Contreras (2004),

d Kumar and Ohana (2008),

e Suzuki and Bekkers (2010b),

f Klausberger and Somogyi (2008),

g Kawaguchi and Kubota (1997),

h Young and Sun (2009),

i Markham et al. (2004),

j Price et al. (2005).

### Pyramidal cell diversity in pars principalis

Pyramidal cells exhibited physiological patterns that distinguished them from the GAD67-GFP cells examined. For example, the projection neurons quickly fired a cluster of two to four action potentials at the start of depolarization followed by a slower regular firing rate (Figures 2D,E). Interestingly, variants of this response were observed on the basis of deep to superficial location in layer II (Figures 2D,E): the initial firing rate for deep cells was even faster than superficial cells (Table 2). Similarly in the APC, pyramidal cells in deeper portions have faster initial firing rates than their more superficial counterparts (Suzuki and Bekkers, 2011). Since there is substantial evidence that neocortical pyramidal cells with different projections have separate morphologies and physiologies (Kumar and Ohana, 2008; Yamashita et al., 2013), that possibility was examined here. Deep vs. superficial AON cells did not vary based upon their projection target, either the OB or PC, and Golgi studies in the rat suggest that they do not differ in morphology (Brunjes and Kenerson, 2010). To date, no one has explicitly compared PC cells with different projections, but it is assumed that neurons in different layers have separate targets (Haberly and Price, 1978).

Pyramidal cells in pars principalis have similar morphological and electrophysiological properties to pyramidal cells in other regions. Table 3 compares cells from the AON, PC, hippocampus, and neocortex. The italicized text highlights differences in how the cell types were classified in each area. The deep-to-superficial differences in pyramidal cell firing pattern are apparent in PC, but not seen in hippocampus or neocortex, perhaps due to the more compact cell layers in those regions. Bursting pyramidal cells, restricted to layers IV and V of neocortex (Connors and Gutnick, 1990), are absent from pars principalis and PC (Suzuki and Bekkers, 2011). The non-bursting subclasses of pyramidal cells in the hippocampus and neocortex are very similar to pars principalis cells in that each exhibits quick initial firing, large action potentials, short and small afterhyperpolarizations. These findings are consistent with the notion that pars principalis is simplified version of more complex areas rather than a cortex with a distinct organization.

### Non-pyramidal cell diversity in pars principalis

The survey of the AON reported above indicated the presence of 13 possible distinct subtypes of GAD67-GFP cells in pars principalis, including cells immunopositive for CB, CR, PV, VIP, SOM, NPY, CCK, CR + PV, PV + CB, CR + VIP, CB + SOM, CR + SOM, and VIP + CCK. The first four of these subtypes each account for more than 10% of the GAD67-GFP cells examined. The results indicate that there are a surprising number of GABAergic phenotypes present in pars principalis, especially in light of the fact that it is a “simple” region compared to neocortex. The AON also contains two unique subtypes (CR + PV, CR + SOM) that are not present in PC. The AON is similar to the PC in that CB, CR, PV, and VIP cells are the most numerous subtypes (Suzuki and Bekkers, 2010a). Both regions slightly differ from the hippocampus and neocortex where the most plentiful cell types are

NPY, CB, PV, and SOM (Jinno and Kosaka, 2006), and PV, CB, and SOM (Kawaguchi and Kubota, 1997), respectively.

The electrophysiological and morphological survey suggested three major classes of non-pyramidal cells: multipolar, neurogliaform, and horizontal. These groups were then further subdivided into four types based upon physiological properties or deep to superficial location: fast and regular spiking multipolar, and superficial and deep neurogliaform cells. Taken together, these five categories are far fewer than the number of subtypes reported in the hippocampus (>20 major classes, Klausberger and Somogyi, 2008) or neocortex (>10; Markham et al., 2004; Ascoli et al., 2008). The anterior PC has the same classes and subtypes as the AON, but with the addition of bifuted cells (Suzuki and Bekkers, 2010a,b). One potential reason for the relative lack of GABAergic cell diversity is that this study is the first to investigate these cells in the AON. Indeed, many of the subtypes uncovered in our immunohistochemical survey were not found in the targeting of GAD67-GFP cells in the patch-clamp study. Further research may elucidate additional cell types.

Table 3 compares classes of non-pyramidal cells in the pars principalis to those most similar in PC, hippocampus, and neocortex. Perhaps not surprisingly, pars principalis cell classes were most similar to those of PC. Some small differences, such as the restriction of multipolar cells to the deep regions in PC (Bekkers and Suzuki, 2013) but not in pars principalis, might be attributed to pars principalis having only one cell body layer. Indeed, the distributions of subtypes of non-pyramidal cells in pars principalis make it appear to be less rigidly structured.

Neurogliaform cells have markedly different distributions in brain regions outside the olfactory cortices. In the hippocampus neurogliaform cells are located in a single deep to superficial location and express NPY (Price et al., 2005). Neurogliaform cells in neocortex are located only in layers II/II and IV, and make-up less than 1% of the non-pyramidal cell population (Markham et al., 2004). Neurogliaform cells were often encountered in this study and were widely distributed, suggesting that they may play a much more significant role in pars principalis' circuitry.

Horizontal cells are the most unique cell type in the olfactory cortices (Table 3). Out of the more than 20 classes of non-pyramidal neurons in the hippocampus, none resemble these cells that are found in layer I of both PC and pars principalis. Cajal-Retzius cells in neocortex have some similarities in terms of morphology and firing pattern, but they are only seen in immature animals (i.e., rats before P10; Hestrin and Armstrong, 1996). Pars principalis does contain a unique variety of this cell class: “vertically oriented” horizontal cells. They might be due to the unique structure of the AON. These cells are often observed where layer II of pars principalis forms a 90° turn at the junction of pars lateralis and pars dorsalis. These cells may emerge in an attempt to maintain an orientation to the sensory inputs in the LOT. Perhaps cells with similar functions but alternative morphologies are located near the input layers in the hippocampus and neocortex. Regardless, the role of horizontal cells in the olfactory system deserves further investigation.

### Diversity in pars externa

Initial observations suggest that cell types in pars externa differed from those in pars principalis. Pars externa is thought to serve as a relay mapping cells with input from one OB glomeruli to its contralateral mirror (Neville and Haberly, 2004; Brunjes et al., 2005; Yan et al., 2008; Kay et al., 2011), and as detector of differences between the OB s (Kikuta et al., 2010). However, the results presented above indicate that pars externa has a surprising amount of cell diversity. All seven individual molecular markers investigated and four colocalized pairs of markers were found in GAD67-GFP cells in pars externa. Additionally, pars externa contains several unique kinds of cells including a large portion of CB-positive projection neurons that do not express GAD-67. If indeed more complex neural computations require more diversity of inhibitory neurons (Klausberger and Somogyi, 2008), the results presented above suggest that pars externa must play a complicated role in olfactory processing.

## Conclusions

The present study is the first of its kind to carefully document the morphological, electrophysiological, and molecular characteristics of the projection neurons and local inhibitory neurons of the AON. Pars principalis contains pyramidal cells and interneurons common in most cortical areas, confirming its cortical nature. When compared to neocortex, the hippocampus and the PC, pars principalis proved to be most similar to the PC; indeed, in several ways it can be viewed as a less complex version of the APC. However, the fact that AON also exhibits cell types not seen in the PC including GAD67 cells expressing both CR and PV, or CR and SOM, multipolar cells located superficially, and vertically-oriented horizontal cells, as well as the previously established differences in connectivity and development (Brunjes et al., 2005) indicate that the two region are functionally distinct. Nevertheless, the apparent simplicity of pars principalis, paired with its well-defined inputs from the OB, make it ideal for investigations of cortical information processing.

### Conflict of interest statement

The authors declare that the research was conducted in the absence of any commercial or financial relationships that could be construed as a potential conflict of interest.
